# Development of a YouthFit Index to assess health-related quality of life in Hong Kong children

**DOI:** 10.1016/j.jped.2025.01.004

**Published:** 2025-02-12

**Authors:** Ka-man Yip, Hung-kwan So, Andy C.Y. Tse, Winnie W.Y. Tso, Parco M.F. Siu, Ian C.K. Wong, Jason C. Yam, Mike Y.W. Kwan, Lobo H.T. Louie, Wilfred H.S. Wong, Patrick Ip

**Affiliations:** a The University of Hong Kong, Department of Paediatrics and Adolescent Medicine, Hong Kong SAR, China; b The Education University of Hong Kong, Department of Health and Physical Education, Hong Kong SAR, China; c The University of Hong Kong, School of Public Health, Hong Kong SAR, China; d Centre for Safe Medication Practice and Research, Department of Pharmacology and Pharmacy, The University of Hong Kong, Hong Kong SAR, China; e Aston School of Pharmacy, Aston University, Birmingham, UK; f The Chinese University of Hong Kong, Department of Ophthalmology and Visual Sciences, Hong Kong SAR, China; g Princess Margaret Hospital, Department of Paediatrics and Adolescent Medicine, Hong Kong SAR, China; h Hong Kong Children's Hospital, Department of Paediatrics and Adolescent Medicine, Hong Kong SAR, China

**Keywords:** HRQoL, Physical fitness, Sleep, Index, MFA, Children

## Abstract

**Objective:**

To develop a YouthFit Index, a comprehensive and easily interpretable measure of youth health-related quality of life (HRQoL).

**Method:**

A repeated cross-sectional design was employed to develop and validate the YouthFIt index using the Pediatric Quality of Life Inventory (PedsQL). Activity level and sleep data were collected by a wearable device, Actigraph. Questionnaires were used to gather demographic information and PedsQL data. Multiple-factor analysis of mixed data was employed to identify principal components (PCs) of the modifiable lifestyle variables. The YouthFit Index was calculated by summing the weighted PCs. Logistic regression models were used to assess the odds ratios for risks associated with the YouthFIt Index.

**Results:**

A total of 1,867 students were recruited for the study. The findings showed that having YouthFit Index scores greater than 5 was significantly associated with a lower risk of identifying with special health care needs. Compared to the group with YouthFit Index scores below or equal to 5, the group with scores above the cut-off demonstrated a significant 43 % reduction in the risk of PedsQL total score. The results also revealed that increased sleep duration and physical activity are associated with better HRQoL, while longer sedentary periods and later bedtimes are linked to worse HRQoL.

**Conclusions:**

The YouthFit Index is a user-friendly tool for assessing the HRQoL of Hong Kong Chinese children between the ages of 6 and 17. It can help raise awareness about healthy quality of life and promote health and well-being among children in Hong Kong.

## Introduction

Health-related quality of life (HRQoL) is important for children as it provides a holistic view of their overall well-being which not only incorporates physical health, but also emotional, social, and mental health.^1^ The importance of lifestyle factors in shaping the HRQoL of children and adolescents has been increasingly recognized.^2^^,^^3^ These factors, including physical activity, sleep, sedentary time, and healthy weight, not only contribute to physical health but also play a crucial role in cognitive, emotional, and social development.^4^ Maintaining a healthy lifestyle not only influences physical health but also has a profound impact on psychosocial well-being, which are major dimensions of HRQoL.^4^ Engaging in physical activity has been associated with reduced symptoms of depression and anxiety, enhanced self-esteem, cognitive function, and academic performance.^5^ Similarly, adequate sleep and earlier sleep timing are beneficial for emotional regulation, social functioning, and overall mental health^6^. The relationship between childhood HRQoL and lifestyle has been studied,^2^^,^^4^ but there is currently no standardized way to assess HRQoL and support the development of healthy lifestyle habits in children and adolescents.

Although the Pediatric Quality of Life Inventory™ (PedsQL) questionnaire can be used to measure HRQoL in children, it may be challenging for the general public to understand this clinical score. HRQoL provides a comprehensive overview of various lifestyle factors that can affect the well-being of children. Previous studies focused on examining the associations between lifestyle factors and the HRQoL.^3^ Instead of evaluating individual lifestyle factors in relation to HRQoL, considering them together in a combined score may be more effective as it summarizes these behaviors and considers the correlations among these modifiable factors.^7^ In addition, most of these studies were based on questionnaires, objective measures on lifestyle habits for children are believed to be more accurate.^8^^,^^9^ Meanwhile, there is a lack of a user-friendly composite index specifically designed to assess the combination of lifestyle factors and the impact on HRQoL in the pediatric population. It is crucial to develop a tool that is easily understandable and accessible to the general public, so that parents, educators, and healthcare professionals can assess and support the development of healthy lifestyle habits in children and adolescents using objective measures. Their overall well-being can be enhanced and their HRQoL can be improved through this action.

Therefore, the present study aims to develop a YouthFit Index, which will incorporate multiple factors relevant to children's lifestyles, including physical activity, sleep patterns, sedentary behavior, and body mass index (BMI), to provide a comprehensive and easily interpretable measure of youth HRQoL. Additionally, it will be specifically designed to assess the relationship between lifestyle factors and HRQoL in children.

## Methods

A repeated cross-sectional design was employed in this 4 years study to develop and validate the YouthFIt index. The study was conducted in accordance with the Declaration of Helsinki and approved by the Institutional Review Board of the University of Hong Kong/Hospital Authority Hong Kong West Cluster (Ref: UW 23–071).

### Participants

Participants were recruited from the five main regions in Hong Kong including Hong Kong Island, West Kowloon, East Kowloon, West New Territories, and East New Territories. Stratified random sampling was employed to randomly select primary and secondary schools from each district. This sampling method was previously used in the local prevalence study, which was published in a peer-reviewed journal.^9^ Parental written consents were obtained prior to the study. Each student and their parent received a package containing questionnaires and a physical activity (PA) monitor (Actigraph wGT3X-BT, Pensacola, Florida, USA). The wearable device has been widely utilized in previous peer-reviewed publications for monitoring PA and sleep in children.^9^^,^^10^

### Measurements

#### Anthropometric measurements

Body height and weight of students in all schools were measured barefoot and in light clothing by trained research staff following a standardized protocol provided by the panel committee. Standing height was measured using a stadiometer (seca 217, UK) to the nearest 0.1 cm. The body weight was measured using body composition monitors (Tanita BC-545 N, Japan) 100*g*.

### Questionnaires

The questionnaires were completed by one of the parents and the students to collect demographic and PedsQL data. Additionally, students were instructed to keep a sleep diary to record their sleep times and wake-up times daily.

### PedsQL

The PedsQL tool is a validated flexible method used to assess HRQoL in both healthy children and adolescents, as well as those with short-term and long-term health conditions.^11^

### Actigraph data

The non-wear time and sleep period were classified based on a previous study.^12^ The physical activity level was calculated using the sum of vector magnitude (VM) in 60 s epochs which were processed using ActiLife software version 6.13.4. Cut-offs of PA and sedentary behavior according to Chandler et al. were used to quantify the time spent in sedentary behavior (VM < 3672 per min), light PA (3672 ≤ VM per min < 9816), moderate PA (9816 ≤ VM per min < 23,628), and moderate-to-vigorous physical activity (MVPA) (VM per min ≥ 23,628).^13^

### Data analysis

The statistical analyses were carried out using R software, version 4.0.3 (R Program for Statistical Computing). BMI was computed by dividing weight in kilograms (kg) by the square of height in meters (m^2^). Participants' BMI was categorized into underweight, healthy weight, overweight, and obese groups based on the International Obesity Task Force age- and sex-specific standards.^14^ The underweight group was defined using international cut-off points that pass through a BMI of 17 at age 18, which aligns with the World Health Organization's standard of thinness. On the other hand, the overweight and obese groups were categorized using cut-off points passing through BMI 25 and 30 at age 18, respectively. The children, except for those in the healthy weight group, were classified as being in the non-healthy weight group.

Descriptive analysis was performed to investigate the demographic characteristics of the students, such as age, sex, anthropometric measurements, variables used for index construction, and PedsQL outcomes. Potential outliers were examined and eliminated if the values deviated by three or more standard deviations from the mean of the group, taking into account age and sex.

### YouthFit Index

HRQoL is a multidimensional concept that encompasses physical, emotional, social, and cognitive well-being. Understanding the factors that contribute to HRQoL in young individuals is crucial for promoting their overall health and well-being. Possible factors of HRQoL were described in a model of a previous study.^15^^,^^16^ The construction of the YouthFit Index included these variables which were separated into three groups: protective factors, risk factors, and demographics. Protective factors included average sleep duration and MVPA time per day on weekdays and weekends. Risk factors included average sedentary time and bedtime per day on weekdays and weekends. Demographic variables included age and BMI status.

Multiple factor analysis (MFA) of mixed data was then used to identify the principal components (PCs) of these variables. MFA is a multivariate analysis method capable of handling both continuous and categorical variables that can be grouped into several groups.^17^ The weights assigned to variables within the same group were equal, giving equal importance to each group. According to Chavent et al. (2014),^18^ the PCs are defined as the following:

(i) $$f_{i}=\;\beta_{0i}+\;\sum_{l=1}^{p+m}\beta_{li}x_{l}$$

Where is the th PC, p is the numbers of numerical variables, m is the total number of levels of categorical variables, $x_{l}$ is the lth column of the original dataset and $\beta_{0i}$ and $\beta_{li}$ are the coefficients of the ith PC obtained from the *MFAmix* function in the *PCAmixdata* package implemented in R version 4.0.3.

Since the PCs calculated can vary from positive to negative and may not be suitable for direct use as an index, min-max normalization and a revised scaling technique were applied. This transformation standardized and rescaled the PCs to values between 0 and 10. Higher scores indicated better performance. The formula used was:

(ii) $$f_{i,scaled}=\;\frac{f_{i}-\;f_{i,min}}{f_{i,max}-\;f_{i,min}}\;\times \;10\;\times \;-1$$

Where $f_{i,min}$ and $f_{i,max}$ are the minimum and maximum values of the PC respectively.

The YouthFit Index was calculated by summing the weighted PCs. The weight assigned to each PC was based on the proportion of variance explained by that PC. The formula for calculating the index is:

(iii) $$YouthFit\;Index=\frac{\sum_{i=1}^{k}\sigma_{i}\times \;f_{i,scaled}\;}{\sum_{i=1}^{k}\sigma_{i}}$$

Where k is the total number of PCs selected, $\sigma_{i}$ is the variance explained by the ith PC.

The YouthFit Index can also be represented as an equation of the original dataset, utilizing equations (i), (ii), and (iii) as shown below:

(iv) $$YouthFit\;Index=\;\frac{-10}{\sum_{i=1}^{k}\sigma_{i}}\sum_{i=1}^{k}\sum_{l=1}^{p+m}\frac{\sigma_{i}}{f_{i,max}-\;f_{i,min}}\beta_{li}x_{l}-\frac{10}{\sum_{i=1}^{k}\sigma_{i}}\sum_{i=1}^{k}\frac{\sigma_{i}}{f_{i,max}-\;f_{i,min}}\beta_{0i}-\frac{10}{\sum_{i=1}^{k}\sigma_{i}}\sum_{i=1}^{k}\frac{f_{i,min}\times \sigma_{i}}{f_{i,max}-\;f_{i,min}}$$

### PedsQL

The total PedsQL scores were classified into risk and normal groups based on the clinically meaningful cut-off established by Huang et al.^19^ Participants who fell into the risk group were potentially identified as having special healthcare needs or chronic conditions.

The YouthFIt Index was subsequently divided into two groups using Receiver Operating Characteristic (ROC) curve analysis in relation to the PedsQL total score. Logistic regression models were utilized to assess the odds ratios (OR) with 95 % confidence intervals (CI) for risks associated with the YouthFIt Index categories with and without adjustment of sex.

## Results

### Participants’ characteristics

A total of 1867 students were recruited for the study during the past 4 years, including 831 primary students and 1036 secondary students. Table 1 provides a summary of the student's characteristics. Among the students, 59.6 % were female, and the average age was 12.4 years. Approximately 76.0 % of the students had a healthy weight. The average time spent in MVPA per day was similar between weekdays and weekends, with 0.50 h and 0.51 h, respectively. However, students had a longer average sedentary time per day on weekdays (11.2 h) compared to weekends (10.3 h). It was observed that students tended to go to bed later during weekends (00:02) compared to weekdays (23:26), but they had a longer sleep duration on weekends (9.6 h) compared to weekdays (7.2 h). The average PedsQL score (78.0 ± 13.3) and the scores for the four scales - physical functioning (82.0 ± 14.3), emotional functioning (70.7 ± 20.3), social functioning (83.1 ± 16.6), and school functioning (75.8 ± 16.4) - closely aligned with the norm recently published in Hong Kong.^2^

**Table 1:** Participant's characteristics.

		N/mean	%/SD
Number of participants		1867	
School type			
Primary school		831	44.5 %
Secondary school		1036	55.5 %
Sex			
F		1113	59.6 %
M		754	40.4 %
Age, years		12.4	2.6
Height, cm		151.6	14.8
Weight, kg		45.7	14.9
BMI		19.4	3.9
BMI Status			
Underweight		54	2.9 %
Healthy weight		1419	76.0 %
Overweight		301	16.1 %
Obese		93	5.0 %
Healthy		1419	76.0 %
Non-healthy		448	24.0 %
Actigraph data			
MVPA time per day, hours	Weekdays	0.5	0.4
MVPA time per day, hours	Weekends	0.5	0.5
Sedentary time per day, hours	Weekdays	11.2	2.1
Sedentary time per day, hours	Weekends	10.3	1.91
bedtime (HH:mm [min])	Weekdays	23:26	54.85
bedtime (HH:mm [min])	Weekends	00:02	67.72
sleep duration per day, hours	Weekdays	7.2	0.99
sleep duration per day, hours	Weekends	9.6	1.8
PedsQL scores			
PedsQL total score		78.0	13.3
Physical functioning		82.0	14.3
Emotional functioning		70.7	20.3
Social functioning		83.1	16.6
School functioning		75.8	16.4
PedsQL total score (risk %)		925	49.5 %
Physical functioning (risk %)		1229	65.8 %
Emotional functioning (risk %)		943	50.5 %
Social functioning (risk %)		673	36.0 %
School functioning (risk %)		539	28.9 %

BMI, body mass index; F; female; M, male, MVPA, moderate-to-vigorous physical activity; PedsQL, Pediatric Quality of Life Inventory.

Non-healthy include those underweight and overweight/obese children.

### YouthFit Index

Table 2 shows the variables used in MFA. The variables were grouped into 3 dimensions: 1) Protective factors including weekday and weekend average MVPA time (WkPA and WdPA) and sleep duration (WkSL and WdSL); 2) Risk factors including weekday and weekend average bedtime (WkBD and WdBD) and sedentary time (WkSD and WdSD); 3) Demographic including age and BMI status. Supplemental Appendices 1 and 2 illustrate the percentage of variance explained by and the squared loadings of variables to each PC. All commonalities of each PC exceeded the minimum cut-off value of 0.25.^20^

**Table 2:** Variables used in the multiple factor analysis with mixed data.

Dimension		Data type	Format
Protective factors	Weekday average sleep duration per day	Numeric	hour
	Weekday average MVPA time per day	Numeric	hour
	Weekend average sleep duration per day	Numeric	hour
	Weekend average MVPA time per day	Numeric	hour
Risk factors	Weekday average sedentary time per day	Numeric	hour
	Weekday average bedtime per day	Numeric	24 hr type in hour
	Weekend average sedentary time per day	Numeric	hour
	Weekend average bedtime per day	Numeric	24 hr type in hour
Somatic	Age	Numeric	Completed age^a^
	BMI Status	Categorical	1 – Healthy 0 – non-healthy

BMI, body mass index; MVPA, moderate-to-vigorous physical activity.

a Completed age: e.g., 6 = 6.00–6.99.

Table 3 presents the coefficients of expression (i). Based on expressions (iii) and (iv), the final formula for calculating the YouthFit Index is as follows:

(v) $$YouthFit\;Index=\;0.2226\;\times f_{1,scaled}+0.3360\;\times f_{2,scaled}+0.4414\;\times f_{3,scaled}=13.2950+0.2361\;\times WdSL+0.2319\;\times WdPA+0.0185\;\times WkSL+0.2161\;\times WkPA-0.0412\;\times WdSD-0.2314\;\times WdBD-0.0405\;\times WkSD-0.1360\;\times WkBD-0.1305\;\times Age+1.4038\;\times Healthy$$

**Table 3:** Coefficients for construction of factor scores.

Variable	PC 1	PC 2	PC 3
(Intercept)	−13.185	−7.701	2.875
Weekday average sleep duration per day	−0.291	−0.261	0.072
Weekday average MVPA time per day	−0.615	0.648	−0.243
Weekend average sleep duration per day	−0.009	−0.177	0.090
Weekend average MVPA time per day	−0.500	0.570	−0.274
Weekday average sedentary time per day	0.105	−0.079	0.022
Weekday average bedtime per day	0.291	0.285	−0.089
Weekend average sedentary time per day	0.104	−0.052	0.000
Weekend average bedtime per day	0.175	0.241	−0.105
Age	0.214	−0.030	0.028
BMI Status: Healthy	0.016	−0.207	−0.516
BMI Status: Non-healthy	−0.049	0.655	1.634

BMI: body mass index, MVPA: moderate-to-vigorous physical activity.

Non-healthy include those underweight and overweight/obese children.

### Association between PedsQL and YouthFit Index

The ROC curve analysis indicated that a cut-off value of 5 is optimal for differentiating between the risk group and the normal group based on the PedsQL total score. Table 4 displays the odds ratios of PedsQL scores falling below the cut-off based on the YouthFit Index group. The findings indicate that having YouthFit Index scores greater than 5 is associated with a significantly lower risk of identifying with special health care needs. Compared to the group with YouthFit Index scores below or equal to 5, the group with scores above the cut-off demonstrated significant reductions in the risk of PedsQL total score by 43 % (CI 31 %–53 %), physical functioning by 44 % (CI 32 %–54 %), emotional functioning by 36 % (CI 23 %–46 %), social functioning by 49 % (CI 38 %–58 %), and school functioning by 27 % (CI 11 %–41 %).

**Table 4:** Odds ratios (OR) of PedsQL scores falling below cut-off by YouthFit Index.

	Index scores	Total	No. at risk	Rate	OR	95 % C.I.	p-values	OR^a^	95 % C.I.	*p*-values
PedsQL total score	≤5	827	472	0.571	1.00 (reference)			1.00 (reference)		
	>5	1040	451	0.434	0.68	(0.59, 0.77)	0.000	0.57	(0.47, 0.69)	0.000
Physical functioning	≤5	827	601	0.727	1.00 (reference)			1.00 (reference)		
	>5	1040	624	0.600	0.67	(0.58, 0.77)	0.000	0.56	(0.46, 0.68)	0.000
Emotional functioning	≤5	827	467	0.565	1.00 (reference)			1.00 (reference)		
	>5	1040	475	0.457	0.74	(0.65, 0.84)	0.000	0.64	(0.54, 0.77)	0.000
Social functioning	≤5	827	371	0.449	1.00 (reference)			1.00 (reference)		
	>5	1040	307	0.295	0.63	(0.55, 0.72)	0.000	0.51	(0.42, 0.62)	0.000
School functioning	≤5	827	267	0.323	1.00 (reference)			1.00 (reference)		
	>5	1040	268	0.258	0.80	(0.69, 0.92)	0.002	0.73	(0.59, 0.89)	0.002

a Adjusted for sex.

## Discussion

In the present study, the authors employed a robust statistical methodology to create and validate a new health-related index for Hong Kong Chinese children between the ages of 6 and 17 using a representative sample. This index is a comprehensive measure incorporating multiple lifestyle factors, including physical activity, sleep patterns, sedentary behavior, and BMI status, which are relevant to children's HRQoL. Traditionally, HRQoL measures based on questionnaires have relied on participant's self-assessments and utilized language that is easily understandable and appropriate for their age. Self-reported questionnaires are vulnerable to several biases, including social desirability and recall bias. These biases can undermine the accuracy and dependability of the data collected. Additionally, children's cognitive development and comprehension abilities can also impact their capability to provide precise responses to questionnaires, further reducing the reliability of self-reported data.^21^ However, the present study sought to go beyond this approach by creating a more comprehensive and objective measure. By incorporating multiple modifiable lifestyle factors into the index to capture a more holistic picture of children's HRQoL. The results revealed that increased sleep duration and MVPA are associated with better HRQoL, while longer sedentary periods and later bedtimes are linked to worse HRQoL.

These findings align with prior research highlighting the significance of healthy lifestyle factors in influencing children's HRQoL. For instance, Wu et al. conducted a systematic review of studies examining the influence of physical activity and sedentary behaviors on HRQoL in children.^3^ They concluded that higher physical activity levels correlated with improved HRQoL, while increased durations of sedentary behavior were associated with lower HRQoL.^3^ Similarly, a recent study in Hong Kong discovered that longer sedentary behavior, shorter sleep duration, and lower MVPA time had negative correlations with HRQoL.^2^

Previous studies looking at HRQoL and health indicators have primarily focused on examining the associations separately. For instance, BMI is a widely used measure to categorize an individual's weight status and identify potential health risks associated with their weight.^22^ Tsiros et al. reported an inverse linear relationship between HRQoL and BMI in a review.^23^ Another example is the International Physical Activity Questionnaire, which assesses the frequency, duration, and intensity of physical activity in individuals, including children.^24^ The level of physical activity in children aged 8 to 14 years is directly linked to their HRQoL.^25^ Children Sleep Habits Questionnaire is a widely used questionnaire to assess the quality of sleep-in children.^26^ Poor sleep quality is associated with impaired HRQoL.^27^ With these considerations, the study provides a more holistic assessment of children's HRQoL. Although these health indices are valuable in assessing specific health aspects or lifestyle behaviors, they do not provide a comprehensive overview of the multiple lifestyle factors in children. Furthermore, those health indicators that depend on questionnaires are time-consuming and challenging to interpret. In contrast, the YouthFit Index is specifically designed using easily measurable factors to assess the combination of lifestyle habits and their HRQoL in this population.

### Implications

The YouthFit Index offers several potential implications and utilities for promoting the health and well-being of children. Firstly, the YouthFit Index is a simple score ranging from 0 to 10 and the cut-off is age-independent, it is easy to be understood by the public and even among children. It serves as a tool for assessment of the overall health of children. With advances in technology, the YouthFit Index can be easily implemented on webpages or mobile apps, making it accessible to a wider audience. Parents, educators, and healthcare professionals can assess children's HRQoL and make necessary adjustments to their lifestyle habits by inputting factors such as physical activity, sleep patterns, sedentary time, age, and weight status. This allows them to track changes in their children's HRQoL over time, making it easier to identify potential issues and take proactive steps to address them using a single index. Secondly, the YouthFit Index can be employed in longitudinal studies to observe children's HRQoL changes over time and serves as a crucial element in designing and evaluating interventions targeting their HRQoL improvement. By offering a comprehensive measure of multiple lifestyle factors' impact on HRQoL, researchers and practitioners can better gauge the efficacy of various strategies in fostering healthy habits and enhancing overall well-being. This insight assists in developing evidence-based programs and policies targeting young people's health and well-being. This can help refine existing programs or create new, targeted tailored interventions to boost children's HRQoL.

### Strength and limitation

One of the main strengths of the present study is the development of a comprehensive index that incorporates multiple lifestyle factors relevant to children's HRQoL. The YouthFit Index incorporated multiple lifestyle factors (physical activity, sedentary behavior, and BMI status) and their impact on children's health-related quality of life. It provides a more holistic view of children's well-being compared to existing indices that focus on single aspects of health or lifestyle behaviors. Another strength of the study is this study is the utilization of objective data obtained from accelerometers worn by the participants, which offers a more reliable assessment compared to self-reported questionnaires. This objective data collection method enhances the reliability and accuracy of the measurements, reducing potential biases and record fatigue associated with parental assessment. Furthermore, the diverse sample of Hong Kong Chinese children between the ages of 6 and 17 adds generalizability to the findings and ensures that the YouthFit Index is applicable to a broad range of children within this population.

However, there are limitations to consider, such as the cross-sectional design, which does not allow for the assessment of causal relationships between lifestyle factors and HRQoL. Future studies employing a longitudinal design could provide valuable insights into the direction and strength of these associations over time. Additionally, the YouthFit Index was developed and validated in a specific population of Hong Kong Chinese children. Although this enhances its applicability to this demographic, it may limit its generalizability to children from other cultural backgrounds or geographical locations. Further research is needed to assess the validity and utility of the YouthFit Index in different populations.

In conclusion, the YouthFit Index is a user-friendly tool for assessing the HRQoL of Hong Kong Chinese children between the ages of 6 and 17. It can help raise awareness about the importance of a healthy quality of life in Hong Kong children. Its application in various settings, from individual assessments to large-scale population studies, may help inform the development of targeted interventions and policies that promote health and well-being among young people, ultimately leading to a healthier society and a more efficient healthcare system.

### Implications and contribution

This study develops a YouthFit Index which is a comprehensive tool for assessing children's health-related quality of life. It can be used for individual assessments or large-scale population studies, aiding the development of interventions and policies that promote well-being among young people and improve healthcare efficiency.

## Conflicts of interest

The authors declare no conflicts of interest.

## Appendix Supplementary materials

Image, application 1

## References

[1] Matza L.S., Swensen A.R., Flood E.M., Secnik K., Leidy N.K. (2004). Assessment of health-related quality of life in children: a review of conceptual, methodological, and regulatory issues. Value Health.

[2] Wong C.K., Wong R.S., Cheung J.P., Tung K.T., Yam J.C., Rich M. (2021). Impact of sleep duration, physical activity, and screen time on health-related quality of life in children and adolescents. Health Qual Life Outcomes.

[3] Wu X.Y., Han L.H., Zhang J.H., Luo S., Hu J.W., Sun K. (2017). The influence of physical activity, sedentary behavior on health-related quality of life among the general population of children and adolescents: a systematic review. PLoS One.

[4] Qin Z., Wang N., Ware R.S., Sha Y., Xu F (2021). Lifestyle-related behaviors and health-related quality of life among children and adolescents in China. Health Qual Life Outcomes.

[5] Rodriguez-Ayllon M., Cadenas-Sánchez C., Estévez-López F., Muñoz N.E., Mora-Gonzalez J., Migueles J.H. (2019). Role of physical activity and sedentary behavior in the mental health of preschoolers, children and adolescents: a systematic review and meta-analysis. Sports Med.

[6] Chaput J.P., Gray C.E., Poitras V.J., Carson V., Gruber R., Olds T. (2016). Systematic review of the relationships between sleep duration and health indicators in school-aged children and youth. Appl Physiol Nutr Metab.

[7] Solera-Sanchez A., Adelantado-Renau M., Moliner-Urdiales D., Beltran-Valls M.R. (2021). Health-related quality of life in adolescents: individual and combined impact of health-related behaviors (DADOS study). Qual Life Res.

[8] Choi L., Liu Z., Matthews C.E., Buchowski M.S. (2011). Validation of accelerometer wear and nonwear time classification algorithm. Med Sci Sports Exerc.

[9] Cole R.J., Kripke D.F., Gruen W., Mullaney D.J., Gillin J.C. (1992). Automatic sleep/wake identification from wrist activity. Sleep.

[10] Sadeh A., Sharkey K.M., Carskadon M.A. (1994). Activity-based sleep-wake identification: an empirical test of methodological issues. Sleep.

[11] Varni J.W., Seid M., Kurtin P.S. (2001). PedsQL 4.0: reliability and validity of the pediatric quality of life inventory version 4.0 generic core scales in healthy and patient populations. Med Care.

[12] So H.K., Chua G.T., Yip K.M., Tung K.T., Wong R.S., Louie L.H. (2022). Impact of COVID-19 pandemic on school-aged children's physical activity, screen time, and sleep in Hong Kong: a cross-sectional repeated measures study. Int J Environ Res Public Health.

[13] Chandler J.L., Brazendale K., Beets M.W., Mealing B.A. (2016). Classification of physical activity intensities using a wrist-worn accelerometer in 8-12-year-old children. Pediatr Obes.

[14] Cole T.J., Bellizzi M.C., Flegal K.M., Dietz W.H. (2000). Establishing a standard definition for child overweight and obesity worldwide: international survey. BMJ.

[15] Haile S.R., Peralta G.P., Raineri A., Rueegg S., Ulytė A., Puhan M.A. (2024). Determinants of health-related quality of life in healthy children and adolescents during the COVID-19 pandemic: results from a prospective longitudinal cohort study. Eur J Pediatr.

[16] Michel G., Bisegger C., Fuhr D.C., Abel T., KIDSCREEN group (2009). Age and gender differences in health-related quality of life of children and adolescents in Europe: a multilevel analysis. Qual Life Res.

[17] Abdi H., Williams L.J., Valentin D. (2013). Multiple factor analysis: principal component analysis for multitable and multiblock data sets. WIREs Computational Statistics.

[18] Chavent M., Kuentz-Simonet V., Labenne A., Saracco J.Ã. Multivariate analysis of mixed type data: the PCAmixdata R package. 2014. ArXiv Prepr. ArXiv14114911.

[19] Huang I.C., Thompson L.A., Chi Y.Y., Knapp C.A., Revicki D.A., Seid M. (2009). The linkage between pediatric quality of life and health conditions: establishing clinically meaningful cutoff scores for the PedsQL. Value Health.

[20] Zeller R.A. (2005). Measurement error, issues and solutions. Encycloped Soc Measurem.

[21] Riley A.W. (2004). Evidence that school-age children can self-report on their health. Ambul Pediatr.

[22] Centers for Disease Control and Prevention (CDC). About child & teen BMI. 2024. [Accessed January 22, 2025]. Available from:https://www.cdc.gov/bmi/faq/?CDC_AAref_Val=https://www.cdc.gov/healthyweight/assessing/bmi/childrens_bmi/about_childrens_bmi.html

[23] Tsiros M.D., Olds T., Buckley J.D., Grimshaw P., Brennan L., Walkley J. (2009). Health-related quality of life in obese children and adolescents. Int J Obes (Lond).

[24] Craig C.L., Marshall A.L., Sjöström M., Bauman A.E., Booth M.L., Ainsworth B.E. (2003). International physical activity questionnaire: 12-country reliability and validity. Med Sci Sports Exerc.

[25] Calzada-Rodríguez J.I., Denche-Zamorano Á.M., Pérez-Gómez J., Mendoza-Muñoz M., Carlos-Vivas J., Barrios-Fernandez S. (2021). Health-related quality of life and frequency of physical activity in Spanish students aged 8-14. Int J Environ Res Public Health.

[26] Owens J.A., Spirito A., McGuinn M. (2000). The Children's Sleep Habits Questionnaire (CSHQ): psychometric properties of a survey instrument for school-aged children. Sleep..

[27] Lee S., Kim J.H., Chung J.H. (2021). The association between sleep quality and quality of life: a population-based study. Sleep Med.

