# Supplementary material for: Development of a YouthFit Index to assess health-related quality of life in Hong Kong children
Source: J Pediatr (Rio J). 2025 Feb 12;101(3):416–23. doi: 10.1016/j.jped.2025.01.004 (PMC12039373; doi:10.1016/j.jped.2025.01.004)
Supplement: Supplementary file 1 [file mmc1.docx]

**JPED-D-24-00252_Supplementary Materials**

**Supplemental Appendix 1** Scree plot for percentage of explained variance by each principal component.

| PC | Eigenvalue | Variance percent | Cumulative variance percent |
| --- | --- | --- | --- |
| 1 | 2.067 | 31.6 | 31.6 |
| 2 | 1.054 | 16.1 | 47.7 |
| 3 | 0.979 | 15.0 | 62.7 |
| 4 | 0.740 | 11.3 | 74.0 |
| 5 | 0.496 | 7.6 | 81.6 |
| 6 | 0.411 | 6.3 | 87.8 |
| 7 | 0.349 | 5.3 | 93.2 |
| 8 | 0.242 | 3.7 | 96.9 |
| 9 | 0.134 | 2.0 | 98.9 |
| 10 | 0.070 | 1.1 | 100.0 |

**Supplemental Appendix 2.** Squared loadings (Cos2) of variables to each PC.

|  | PC 1 | PC 2 | PC 3 | communality |
| --- | --- | --- | --- | --- |
| Weekday average sleep duration per day | 0.490 | 0.201 | 0.014 | 0.705 |
| Weekday average MVPA time per day | 0.361 | 0.204 | 0.027 | 0.591 |
| Weekend average sleep duration per day | 0.002 | 0.318 | 0.076 | 0.395 |
| Weekend average MVPA time per day | 0.323 | 0.214 | 0.046 | 0.583 |
| Weekday average Sedentary time per day | 0.315 | 0.091 | 0.006 | 0.412 |
| Weekday average bedtime per day | 0.482 | 0.237 | 0.021 | 0.740 |
| Weekend average Sedentary time per day | 0.260 | 0.033 | 0.000 | 0.293 |
| Weekend average bedtime per day | 0.283 | 0.273 | 0.048 | 0.605 |
| Age | 0.641 | 0.006 | 0.005 | 0.653 |
| BMI Status | 0.002 | 0.146 | 0.846 | 0.994 |

BMI, body mass index; MVPA, moderate-to-vigorous physical activity.
